# CFTR Modulators: Does One Dose Fit All?

**DOI:** 10.3390/jpm11060458

**Published:** 2021-05-24

**Authors:** Renske van der Meer, Erik B. Wilms, Harry G.M. Heijerman

**Affiliations:** 1 Department of Pulmonology, Haga Teaching Hospital, Els Borst-Eilersplein 275, 2545 AA The Hague, The Netherlands; 2 Central Hospital Pharmacy, Charlotte Jacobslaan 70, 2545 AB The Hague, The Netherlands; e.wilms@ahz.nl; 3 Department of Pulmonology, University Medical Center Utrecht, Heidelberglaan 100, 3584 CX Utrecht, The Netherlands; h.g.m.heijerman@umcutrecht.nl

**Keywords:** cystic fibrosis, CFTR modulators, pharmacodynamics, pharmacokinetics, exposure response relationship

## Abstract

For many people with cystic fibrosis (pwCF), CFTR modulators will be the cornerstone of their treatment. These modulators show robust treatment effects at group level in pwCF with specific mutations. The individual effect however, is variable. In this review we will explain reasons for reconsideration of dosing regimens of CFTR modulating therapy in order to improve treatment response and prevent side effects. Since the effect of a drug depends on pharmacodynamics and pharmacokinetics, pharmacodynamics and pharmacokinetic properties of CFTR modulators will be discussed. Pharmacokinetic-pharmacodynamic relationships will be used to gain insight in dosage response and exposure response relationships. To understand the cause of variation in drug exposure, pharmacokinetic properties that may change due to CF disease will be explained. We show that with current insight, there are conceivable situations that give reason for reconsideration of dosing regimens, however many questions need to be unravelled.

## 1. Introduction

Cystic fibrosis (CF) is a chronic, hereditary, multi-organ disease caused by absence or dysfunction of the cystic fibrosis transmembrane conductance regulator (CFTR) protein [1]. Over the past decade, CFTR protein modulators have been developed, which improve CFTR function either through potentiation of the abnormal protein channel at the cell surface (ivacaftor), or through correction of protein transport to the cell surface (lumacaftor, tezacaftor, elexacaftor). These treatments have now been approved by the European Medicines Agency and US Food and Drug Administration for use in people with CF (pwCF) and specific mutations. With the development of the CFTR modulators, a new era in CF treatment has arrived. Recent trials show an impressive clinical effect of combination therapy with elexacaftor plus tezacaftor plus ivacaftor. Heijerman et al. showed an increase in forced expiratory volume in one second (FEV1) of 10 percentage points in patients homozygous for the Phe508 del mutation ([95% CI 7.4 to 12.6], *p* < 0.0001) after 4 weeks of treatment with elexacaftor/tezacaftor/ivacaftor compared to tezacaftor/ivacaftor [2]. Moreover, elexacaftor/tezacaftor/ivacaftor was shown to be efficacious in pwCF with phe508del-minimal function (MF) genotypes, in whom previous CFTR modulator regimens were ineffective. For this genotype, Middleton et al. showed a 13.8 points higher ppFEV1 at 4 weeks and 13.4 points through week 24 compared to placebo [3]. Recently, the FDA approved elexacaftor/tezacaftor/ivacaftor in pwCF of 12 years and older with at least one of 177 newly approved-mutations other than phe508del. The results are promising and show the potential of life changing improvements for these patients.

For many pwCF, CFTR modulators will be the cornerstone of their treatment. Although these modulators show robust treatment effects at group level, the individual effect is variable [2,3,4,5]. In the supplementary figures, Wainwright et al. showed figures a change from baseline in ppFEV1 ranging from −10% to >+10% after 24 weeks of treatment with lumacaftor/ivacaftor [4]. Heijerman et al., showed the individual repsonse to elexacaftor/tezacaftor/ivacaftor treatment in the supplementary figures, with a change in ppFEV1 ranging from −2.5% to >+20% [2]. The effect of a drug in an individual patient is a result of what the drug does to the body (pharmacodynamics) and what the body does to the drug (pharmacokinetics). We will briefly mention pharmacodynamic properties of CFTR modulators but an extensive explanation of pharmacodynamics is out of the scope of this review. In order to gain insight in the degree to which the drug dosage influences the treatment effect, we will review data on exposure response relationship of these drugs first. Second, we will focus on pharmacokinetic principles and features of CF disease that may contribute to variation in drug exposure.

Registered dosing recommendations of CFTR modulators are based on pharmacodynamic effects in vitro, serum pharmacokinetic studies, and early dose escalating (phase II) studies. In the final part of this review we will discuss remaining questions which need to be resolved in order to determine if current dosing strategies can be applied to all patients or need reconsideration in certain patient groups. 

## 2. Pharmacodynamics of CFTR Modulators

Mutations in the CFTR gene lead to dysfunction of the CFTR ion channel [6]. A group of drugs named CFTR modulators, have been developed to improve this function. Ivacaftor, currently the only approved CFTR potentiator, facilitates increased chloride transport by potentiating the channel-open probability (or gating) of the CFTR protein at the cell surface [7]. Currently three correctors entered the market: First lumacaftor, a first generation CFTR corrector, acts directly on phe508del-CFTR to improve its cellular processing and trafficking, thereby increasing the quantity of functional CFTR at the cell surface [4]. Second, tezacaftor, a second generation CFTR corrector that binds to the first Membrane Spanning Domain (MSD-1) of CFTR and has the same mechanism of action as lumacaftor [5,8]. Thirdly, elexacaftor, a next generation corrector that binds to different sites on the CFTR protein compared to tezacaftor, leading to an additive effect in facilitating the cellular processing and trafficking of phe508del-CFTR and thereby increasing the amount of CFTR protein delivered to the cell surface.

The exact mechanisms by which lumacaftor, tezacaftor and elexacaftor improve cellular processing and trafficking of phe508del-CFTR, and ivacaftor potentiates phe508del-CFTR are not known. The impact of the CF airway environment on these mechanisms remains unclear. Interestingly, a recent study showed beneficial effects of inflammation on CFTR rescue by modulator therapy in vitro [9]. 

Ivacaftor is registered as monotherapy for specific gating mutations in the CFTR gene, or in combination with lumacaftor (for patients homozygous for phe508del mutation), in combination with tezacaftor (for patients homozygous for phe508del mutation or phe508del mutation and specific residual function mutations), and in triple combination with both elexacaftor and tezacaftor (for patients with at least one phe508del mutation) [10]. 

Dose response relationships of currently registered CFTR modulators were investigated in phase II studies in adult pwCF with specific genotypes. Pharmacodynamic endpoints, e.g., FEV1, nasal potential difference (NPD), and sweat choride were measured and compared between study groups with escalating dosing regimens. For ivacaftor, lumacaftor and tezacaftor monotherapy, a trend of increasing response with higher dose was observed [11,12,13]. A range of doses for ivacaftor in combination therapy was not studied. For elexacaftor combined with tezacaftor and ivacaftor, no clear dose response has been seen, as the 100 mg arm showed a response lower than the 50 mg and 200 mg. These results could suggest a rather flat dose-response curve or a maximum effect at a dose level below the tested dosages [14]. 

## 3. Exposure Response Relationship

Dosing regimens of approved CFTR modulators are based on pharmacodynamic effects in vitro (data not published), serum pharmacokinetic studies, and early dose ranging (phase II) studies. Robust treatment effects of CFTR modulating therapies in pwCF with specific genotypes have been demonstrated [2,3,4,5]. However, high variability in treatment response has been observed in individual patients with the same genotype and treatment dosage [2,4]. The question is whether the same dosage of CFTR modulators results in differences in drug exposure and thereby variation in treatment response. In this chapter we will focus on the importance of understanding the exposure response relationship of CFTR modulators, we will discuss what is currently known, and propose methods to investigate exposure response relationships.

### 3.1. Importance of Understanding Exposure Response Relationships

Among various pwCF, high variability in clinical response to CFTR modulating therapy has been observed [4,5,8,15]. The underlying causes of different drug responses and clinical outcomes might be partially attributed to variation in drug exposure. In this context, knowledge of the therapeutic window is important. The therapeutic window (or pharmaceutical window) of a drug is the range of drug concentrations which can treat disease effectively without having toxic effects. In clinical trials, CFTR modulators were generally well-tolerated, with the exception of lumacaftor/ivacaftor which showed a higher rate of respiratory-related adverse events [4]. Observational studies with real-world CFTR modulator safety data however, have shown higher rates of discontinuation as well as adverse events that were rarely observed and not described in the clinical trial setting [16]. Regarding the therapeutic window of ivacaftor it is important to mention data from several studies in target tissues reporting destabilization of corrected phe508del CFTR by too high ivacaftor concentrations, dramatically increasing its turnover rate [17,18,19]. Chronic ivacaftor treatment also reduced mature wild-type CFTR levels and function [17,18,19]. This suggests that a too high ivacaftor exposure can do harm. This underlines the importance of knowledge of the exposure effect relationship of this drug. These findings also demonstrate that chronic treatment with CFTR potentiators and correctors may have unexpected effects and may require optimization of dosing regimens.

### 3.2. Exposure Response Relationship of CFTR Modulators, What Do We Know?

The results of an exposure-response analysis for ivacaftor can be found in the FDA report [11]. Phase II studies showed no additive effect of ivacaftor dosage 250 mg q12h over 150 mg q12h. A direct maximal effect (Emax) model was used to define the relationship of FEV1 and sweat chloride with ivacaftor exposure in pwCF. Ivacaftor dose of 150 mg q12h was selected based on simulations showing that this dose would be needed to achieve an average steady state ivacaftor trough concentration (Cmin,ss) of at least the estimated concentration at which the effect is at 90% of the maximum (EC90) for FEV1 endpoint and 84% (EC84) value for sweat chloride endpoint. This trough concentration was estimated to be approximately 0.25 µg/mL. As shown in Table 1 mean (SD) Cmin of ivacaftor is above this level, 0.8 (0.3) µg/mL. Because no specific dose-limiting safety concerns were identified in early dose escalation studies no exposure-response analysis for safety was performed. However, in daily practice side effects in patients on ivacaftor treatment have been observed, which arises questions about the potential concerns of too high ivacaftor exposure [16].

Data in the FDA report [12] for lumacaftor/ivacaftor show a greater reduction in sweat chloride with increasing lumacaftor concentrations and a slight increase in effect with the addition of ivacaftor. EC50 of lumacaftor for sweat chloride was estimated at trough levels of 4.5 µg/mL. For tezacaftor the average concentration for 50% of the maximum effect (EC50) was 0.5 µg/mL for sweat chloride and 0.4 µg/mL for ppFEV1. No data about target levels of ivacaftor, other than the slightly increase of Emax (for sweat chloride and ppFEV1) by adding ivacaftor to tezacaftor, are mentioned [13]. An in vitro study in phe508del/phe508del and phe508del/MF human bronchial epithelial cells, shows that elexacaftor enhances the chloride transport depending on concentration, with a larger effect than achieved by tezacaftor/ivacaftor. EC50 for elexacaftor in combination with tezacaftor/ivacaftor has been estimated in vitro but no in vivo data are available [14].

Due to development of new CFTR modulators, many pwCF have changed their CFTR modulator regimen. For CF patients homozygous for the F508 mutation currently three CFTR modulator regimens are approved by the FDA and EMA. Many patients have now switched from lumacaftor/ivacaftor to tezacaftor/ivacaftor or more recently, to elexacaftor/tezacaftor/ivacaftor. In several patients, we observed differences in clinical outcome and tolerability after changing lumacaftor/ivacaftor to tezacaftor/ivacaftor. This arises questions about changes in drug exposure when switching from one modulator regimen to another. We have measured steady state trough levels of lumacaftor and ivacaftor in 24 adult CF patients who planned to switch to tezacaftor/ivacaftor and we measured trough levels of tezacaftor and ivacaftor in steady state in the same patients. Although tezacaftor/ivacaftor treatment contains a lower dose of ivacaftor compared to the lumacaftor/ivacaftor (300 vs. 500 mg/day), ivacaftor trough concentrations were seven times higher after tezacaftor/ivacaftor treatment compared to lumacaftor/ivacaftor treatment (mean 7.08, range 1.12–34.30; *p* = 0.00 (Wilcoxon), unpublished data). If this increased exposure to ivacaftor observed within individual patients is clinically relevant needs to be elucidated. 

### 3.3. How to Obtain Insight in Exposure Response Relationship of CFTR Modulators

#### 3.3.1. Plasma and Cellular Drug Concentrations

For drugs such as CFTR modulators that act within cells, intra cellular concentrations would ideally be obtained to be related to treatment effect. Peripheral blood however, is easily accessible and would allow to monitor the pharmacokinetic profile of CFTR modulator treatment at patient level. Analytical methods have been developed and validated for rapid detection and quantification of ivacaftor, its major metabolites, lumacaftor and tezacaftor in the plasma and sputum of pwCF [20,21]. Guimbellot et al. observed a correlation between plasma and cellular ivacaftor concentrations, but cellular concentrations were disproportionally more elevated in patients with higher plasma concentrations [22]. This suggests in vivo accumulation of ivacaftor, which has also been mentioned in in vitro reports [23]. The higher cellular concentrations may result in a level of CFTR restoration distinct from what would be expected from plasma concentrations.

#### 3.3.2. Organoids

Plasma samples from CFTR modulator-treated CF patients have been used to personalize pharmacokinetics and pharmacodynamics by organoid testing. The primary readout (forskolin-induced swelling or FIS) is CFTR dependent, and there is evidence for a correlation between the modulator-induced FIS response and the change in FEV_1_ and sweat chloride concentration in vivo [24]. Dekkers et al. described a bioassay to measure CFTR modulator activity in human plasma using intestinal organoids. They observed a dose-dependent increase of forskolin-induced organoid swelling for ivacaftor [25]. More recently, other techniques with spheroids formed from nasal and bronchial epithelial cells and two- and three-dimensional nasal cultures have been developed, in order to better characterise new mutations and to guide therapy optimization [26,27]. These organoid assays may help us to better understand the relationship between drug exposure and treatment response. 

## 4. Pharmacokinetics of CFTR Modulators and CF Features That May Change Pharmacokinetic Properties

Pharmacokinetics show what the body does to the drug. Different features of CF disease may influence pharmacokinetic properties of drugs which may contribute to variation in drug exposure. In order to gain insight in the pharmacokinetics of a certain drug, we will explain four main pharmacokinetic processes: absorption, distribution, metabolism and excretion [28] with a focus on CFTR modulators. 

It is already known that certain drugs have altered pharmacokinetic properties in pwCF compared to non-CF subjects [29,30]. Although little is known about the intersubject variability in the CF population, some studies have been published showing differences between CF patients in clinical pharmacokinetic parameters of several drugs [31,32,33]. With the development of highly effective modulators, the CF population may even become more heterogenic and differences in pharmacokinetic properties within the CF population itself (interpatient variability) may become more important. Moreover, pharmacokinetic properties of certain drugs may alter after restoring CFTR function in pwCF who are starting treatment with highly effective modulators, which may increase intrapatient variability. In this chapter we will discuss features of CF disease that may change pharmacokinetic properties and thereby may cause inter- and intrapatient variability in drug exposure. 

### 4.1. Oral Absorption

#### 4.1.1. Basic Principles

Absorption is defined as how the drug moves from the site of administration to the prehepatic bloodstream. Absorption and the consequent first hepatic passage defines bioavailability (the fraction of the drug that reaches the systemic circulation). Orally administered medication may have variable bioavailability which depends on several factors including the disintegration and dissolution of solids, gastric emptying rate, dietary content, first hepatic pass effect, presence of interacting medication and the acidity of gastric contents. 

#### 4.1.2. Oral Absorption of CFTR Modulators

Administered as an oral dose, CFTR modulators are absorbed directly from the gut. All CFTR modulators should be taken with fat containing food because the bioavailability of ivacaftor, lumacaftor and elexacaftor increases by two to four times compared to a fasting state [34,35,36]. The exposure of tezacaftor does not change when given with a fat meal [37]. Data of maximal concentration (C_max_) and time to maximal concentration (T_max_) for all registered CFTR modulators are shown in Table 1. 

#### 4.1.3. CF Characteristics That Affect Absorption

Drug absorption in patients with CF can be affected by alterations in several factors which we will discuss here [38,39].

Gastro and intestinal transit time: CFTR dysfunction in the intestines causes a decreased water secretion resulting in thick viscous intestinal content with a high risk of intestinal obstruction and delayed transit [39,40]. Further, gastroparesis is a common problem seen in CF patients, especially in patients with poorly controlled cystic fibrosis related diabetes (CFRD) [41]. This delayed motility may contribute to a decreased absorption rate for certain drugs. 

Pancreatic insufficiency: A severe CFTR gene mutation in both alleles results in little or no CFTR chloride channel activity and destruction of the exocrine pancreas [42]. This exocrine pancreatic enzyme deficiency impairs the absorption of dietary fats and lipid-soluble nutrients [43]. Around 85% of pwCF develop exocrine pancreatic insufficiency and despite treatment with pancreatic enzymes patients still suffer from fat malabsorption [44]. This may result in a decreased and delayed absorption of oral drugs. The influence of exocrine pancreatic insufficiency and the effect of treatment with pancreatic enzymes was studied by Dickinson et al. They showed that although pancreatic enzyme replacement improved the absorption characteristics of the chloramphenicol-P formulation, absorption remained prolonged and unreliable. They also showed that exocrine pancreatic insufficiency causes a decreased exposure to drugs that need pancreatic enzymes for the liberation of their active form, e.g., chloramphenicol [45].

Increased bile acid excretion and duodenal hyperacidity: Another gastro intestinal complication in CF is an increased fecal bile acid (BA) excretion [46,47]. In the physiological situation the enterohepatic circulation of BAs is a tightly regulated system in which around 95% of total BAs is reabsorbed and the remaining 5% is excreted via the feces. BAs are important for digestion and absorption of fat- and fat-soluble vitamins. Theoretically this BA dysfunction may play a role in decreasing the exposure to lipophilic drugs in CF patients. CFTR dysfunction is related to postprandial hyperacidity of the duodenum which is caused by increased gastric acid secretion and decreased bicarbonate secretions in the intestine [48,49]. This acidic environment may decrease drug absorption in CF patients. 

When treatment with CFTR modulators is started in an early stage of the disease, organ function may improve. CF patients with pancreatic insufficiency may become pancreas sufficient and thereby drug absorption and exposure may increase. [50,51].

### 4.2. Distribution

#### 4.2.1. Basic Principles

Distribution is the movement of a drug from the systemic circulation to the tissues. Distribution occurs most rapidly into body compartments with a high blood flow (lung, liver, brain). If the volume of distribution which is calculated from plasma concentrations is larger than the body volume, accumulation in plasma cells or tissues occurs. Major factors affecting distribution of drugs are diffusion rate, affinity of the drug to the tissue, perfusion, and binding to plasma proteins. This plasma protein binding (often to albumin) is often reversible and can act as a reservoir. High plasma protein binding results in a lower volume of distribution (Vd) (the amount of drug administered divided by the plasma concentration of that drug). For drugs with a high extravascular binding or storage in fat or other tissues, the volume of distribution is high. 

#### 4.2.2. Distribution of CFTR Modulators

All CFTR modulators are transported in the plasma highly bound (99%) to plasma proteins to their site of action, which is the apical membrane of epithelial cells [34,35,36,37]. Volume of distribution for all registered CFTR modulators are shown in Table 1. 

#### 4.2.3. CF Characteristics That Affect Distribution

CF patients are at risk for malnutrition due to malabsorption, increased energy expenditure and a reduced food intake. Because many pwCF weigh less than healthy subjects but have a relatively higher lean body mass/fat free mass, the extracellular volume of an underweight CF patient will be underestimated when only total bodyweight is taken into account [27]. A higher volume of distribution in CF patients for some drugs can still be found after correction for body composition [52]. This may be caused by an increased total body blood volume and hypoalbuminemia which theoretically may lead to decreased protein binding. This hypoalbuminemia is associated with liver disease, cachexia and inflammation, problems often seen in CF patients [53].

With the introduction of highly effective modulators we expect differences in body composition between and within patients to become more prevalent [54].

### 4.3. Metabolism

#### 4.3.1. Basic Principles

One goal of metabolization is to make the drug easier to excrete. The enzymes involved in metabolism are present in many tissues but mainly in the liver [28]. It involves enzymes that convert prodrugs to active metabolites or convert active drugs to inactive or excretable forms. The liver’s primary mechanism for metabolizing drugs is via a specific group of cytochrome P-450 enzyme, a microsomal superfamily of isoenzymes that catalyzes oxidation and hydroxylation of many drugs. CYP450 enzymes can be induced or inhibited by many drugs and substances. 

Drug metabolism rates vary among patients and are influenced by genetic factors, coexisting disorders (particularly chronic liver disorders and advanced heart failure), and drug interactions (especially those involving induction or inhibition of metabolism).

#### 4.3.2. Metabolism of CFTR Modulators

Ivacaftor, tezacaftor and elexacaftor are extensively metabolized in the liver mainly by cytochrome P450 3A (CYP3A), including both CYP3A4 and CYP3A5. Lumacaftor however, is not extensively metabolized in humans and the majority of lumacaftor is excreted unchanged in the feces. M1 and M6 are the two major metabolites of ivacaftor in humans. M1 is considered pharmacologically active [34]. Administered together with lumacaftor, the steady-state exposure of ivacaftor is decreased due to the CYP3A inducing effect of lumacaftor [35]. M1-TEZ, M2-TEZ, and M5-TEZ are the three major circulating metabolites of tezacaftor in humans. M1-TEZ has similar potency to that of tezacaftor and is considered pharmacologically active, M2-TEZ is much less pharmacologically active and M5-TEZ is not considered pharmacologically active [37]. M23-ELX is elexacaftor’s only major circulating metabolite and is considered pharmacologically active with similar potency to elexacaftor [36].

#### 4.3.3. CF Characteristics That Affect Metabolism

The capacity of the liver to metabolize drugs depends on hepatic blood flow and liver enzyme activity. Factors that may change hepatic metabolism depend on the kind of drug. Drugs with a low hepatic extraction ratio are not sensitive to liver blood flow changes. The fraction of these drugs removed from the blood during a single passage through the liver (the extraction ratio) is small, so their clearance mainly depends on the activity of drug metabolizing enzymes. 

Hepatic metabolism in pwCF may differ due to altered liver enzyme activity and or/changes in liver blood flow. 

Changes in liver enzyme activity: Enhanced hepatic metabolism in CF patients compared to healthy people has been described and may be caused by selective up-regulation of certain enzymes (e.g., cytochrome P450) [29]. However, more recent studies show the opposite, with non-altered CYP1A2, CYP2D6, xanthine oxidase and N-acetyltransferase activities and no increase of CYP3A4 expression in the gut in children with CF [55,56]. Others investigated the association of infection and inflammation, which are common characteristics of CF disease, with a lower expression and activity of hepatic drug-metabolising enzymes (e.g., CYPs) [57,58].

With increased life expectancy, which is in part due to better treatment options, the burden of pharmacotherapy in CF patients will increase, resulting in a higher risk for drug-drug interactions. An example of a common drug interaction is co-treatment of a CYP3A4 inhibitor (e.g., azoles) with a CYP3A4 inducer (e.g., cyclosporine) [59]. Drug interactions with CFTR modulators may also occur during co-administration with certain antibiotics (e.g., clarithromycin, a CYP3A4 inhibitor) [34,35,36,37]. These interactions complicate the interpretation of hepatic metabolism and its influence on expected drug exposure. Therefore, for several drugs (e.g., azoles, immunosuppressants), therapeutic drug monitoring is currently advised. 

Changes in liver blood flow: Liver disease in CF is a common problem [60] and can alter the kinetics of certain drugs [61]. Liver blood flow can be reduced because of pathological alterations caused by liver disease, as in cirrhosis. There can be spontaneous porta-caval shunts. For drugs with a high first pass effect, the shunt may result in the drug bypassing the liver and reaching the systemic circulation directly. This results in increased systemic availability of the drug. 

As already mentioned, CFTR modulators are substrates of CYP3A4 and CYP3A5. Due to interaction with CYP3A4/5 inhibitors or inducers, co-administration is not recommended or requires dose adjustment as is incorporated in the SmPC’s of CFTR modulators. As therapeutic target ranges of CFTR modulators are currently unclear, therapeutic drug monitoring is not (yet) feasible in clinical practice. 

### 4.4. Elimination

#### 4.4.1. Basic Principles

The kidneys are the principal organs for excreting water-soluble substances. The biliary system contributes to excretion to the degree that the drug is not reabsorbed from the gastrointestinal tract. Important principles in understanding elimination are clearance (the rate of elimination of the drug from the body and is the product of the elimination rate constant and the volume of distribution) and half clearance time (the time required for the amount of drug present to be reduced by 50%). 

#### 4.4.2. Elimination of CFTR Modulators

Following oral administration, the majority of ivacaftor, tezacaftor and elexacaftor is excreted in the feces after metabolic conversion (88, 72, 87% respectively). For lumacaftor, the majority (51%) is excreted unchanged in the feces. For ivacaftor, lumacaftor and elexacaftor urinary excretion is negligible, whereas 14% of tezacaftor is excreted in the urine. T_1/2_ and clearance values for all registered CFTR modulators are presented in Table 1. 

#### 4.4.3. CF Characteristics That Affect Elimination

In patients with CF, enhanced renal clearance has been observed for some drugs. In contrast to renal and hepatic clearance, biliary excretion might be decreased in CF [62]. Biliary disorders, prevalent in the CF population, could explain this phenomenon, but more research is needed to confirm this hypothesis.

CFTR modulators are mainly eliminated via feces and not with urine (tezacaftor only, 14%). Impairment of renal function is therefore not likely to change the elimination of CFTR modulators. 

All four pharmacokinetic mechanisms will affect the exposure to the drug. The exposure (AUC) of the approved CFTR modulators in steady state, is shown in Table 1. 

## 5. Conclusions and Future Perspectives

At group level CFTR modulators have shown robust treatment effects in pwCF with specific mutations. Results of both ivacaftor and elexacaftor/tezacaftor/ivacaftor therapy are impressive [2,3,7]. However, treatment effects differ between individual CF patients with similar genotypes. In this review we wanted to give insight in reasons for reconsideration of dosing regimens of CFTR modulating therapy in order to improve treatment response and prevent side effects. Knowledge about pharmacodynamics and pharmacokinetics and finally PK-PD relationships of CFTR modulators is therefore needed. 

For ivacaftor, lumacaftor and tezacaftor a trend of increasing treatment response with higher dose was observed in phase II and III studies. [11,12,13]. To evaluate if differences in treatment effects between patients treated with the same dosage may be caused by difference in exposure, knowledge about the therapeutic window of these drugs is needed. Exposure to ivacaftor was linearly correlated with response, and maximal effect concentration for FEV1 (EC90) and sweat chloride (EC84) was 0.25 µg/mL [10]. The maximal effect concentrations (EC50) of lumacaftor and tezacaftor were estimated at trough levels of 4.5 and 0.5 µg/mL respectively. Maximal effect concentrations of ivacaftor as part of combination therapy have not been investigated. 

In Table 1 we summarized pharmacokinetic parameters from different CFTR modulators. C_min,ss_ of ivacaftor, lumacaftor and tezacaftor is higher than the estimated maximal effect concentrations. Remarkably, C_min,ss_ of ivacaftor as part of lumacaftor/ivacaftor is a factor 10 lower than C_min,ss_ of ivacaftor as part of tezacaftor/ivacaftor treatment and ivacaftor monotherapy (Table 1). The sponsor suggested that ivacaftor potency is seven-fold higher in phe508del-CFTR (EC90 at 0.06 µg/mL) compared to G551D-CFTR (EC90 at 0.4 µg/mL) in the in vitro studies. However, this does not explain the difference with tezacaftor/ivacaftor since both are registered for F508 del homozygous mutations [12]. This arises questions about the ivacaftor dosage as part of combination therapy with CFTR correctors. 

Features of CF disease which may change pharmacokinetic properties and thereby may affect drug exposure were explained. The influence of several conditions such as renal or hepatic impairment, body weight, and drug-drug interactions on drug exposure have already been investigated. Recommended dose adjustments can be found in the SmPC’s of different CFTR modulators. Currently we are performing a study to investigate the influence of pancreatic function on the absorption and exposure to ivacaftor in CF patients. The influence of other patient characteristics, e.g., body composition on the exposure to CFTR modulators needs further investigation. 

Although with current insight, there are conceivable situations that give reason for reconsideration of dosing regimens, writing this review raises many questions that need to be unravelled. 

Data from several in vitro studies showed destabilization of corrected phe508del CFTR and reduction of mature wild-type CFTR levels and function by too high ivacaftor concentrations [17,18,19]. Further research is needed to elucidate if this effect also occurs in vivo and to determine the ivacaftor concentrations giving rise to these negative effects. Organoid models may be helpful to determine the maximal effect concentration of ivacaftor (lower bound of therapeutic window) and to get insight in the concentration above which the negative effect on the corrector takes place (upper bound of therapeutic window). 

In vitro studies have been performed to improve the insight in pharmacokinetic and dynamic properties of CFTR modulators by investigating cellular concentrations of ivacaftor and measuring serum levels [22,23]. More studies on plasma concentrations of CFTR modulators are needed to detect interindividual differences, interactions, and to be able to relate exposure to clinical efficacy and side effects. Besides studies on the relation between plasma and tissue concentrations, investigation of concentration effect relationship in organoids can be helpful to define a therapeutic window. 

## Figures and Tables

**Table 1:** Steady state pharmacokinetic parameters for CFTR modulators in a fed state in patients with CF aged 12 years and older. (data from SmPCs and FDA reports).

	Cmax Mean (SD)	Tmax Median (Range)	Vd Mean (SD)	T1/2 Mean (SD)	Clearance Mean (SD)	AUC Mean (SD)	Cmin Mean (SD)
	µg/mL	h	L	h	L/h	μg∙h/mL	µg/mL
Iva 150 mg q12h *	1.5 (0.6) ng/mL	4 (1–6)	353 (122) #	14.4 (3.9)	17.3 (8.4)	12.9 (3.6)	0.8 (0.5)
Lum 400 mg q12h	25.0 (7.7)	4 (2–9)	86.0 (69.8)	25 (9.9)	2.4	198 (64.8)	9.8 (4.8)
Iva 250 mg q12h	0.6 (0.3)	4 (2–6)	201	9 (3.8)	25.1	3.7 (2.3)	0.08 (0.02)
Tez 100 mg q24h	6.5 (1.8)	4 (2–6)	271 (157)	156	1.3 (0.4)	82.7 (23.3)	1.6
Iva 150 mg q12h	1.3 (0.4)	6 (3–10)	206 (82.9)	9	15.7 (6.4)	10.9 (3.9)	0.7
Elex 200 mg q24h	9.2 (2.1)	6 (4–12)	53.7 (17.7)	25	1.2 (0.3)	162 (47.5)	5.5 (2.7)
Tez 100 mg q24h	7.7 (1.7)	3 (2–4)	82.0 (22.3)	60	0.8 (0.1)	89.3 (23.2)	2.1(0.8)
Iva 150 mg q12h	1.2 (0.3)	4 (3–6)	293 (89.8)	13	10.2 (3.1)	11.7 (4.0)	0.8 (0.3)

Iva = ivacaftor, Lum = lumacaftor, Tez = tezacaftor, Elex = elexacaftor; SD: Standard Deviation (SD for clearance of lum/iva and Vd of iva as lum/iva combination and Cmin of tez/iva were not shown in FDA reports); Cmax: maximum observed concentration; Tmax: time to maximum observed concentration; Vd: volume of distribution; T1/2: terminal half-life; AUC: area under the concentration versus time curve. * ivacaftor steady state data presented show the average of study results in healthy volunteers shown in the FDA report (steady state data for pwCF and healthy volunteers are comparable as mentioned in the FDA report, reference ID: 3073639). # data after single dose.

## Data Availability

Not applicable.
